# On the Age of Leprosy

**DOI:** 10.1371/journal.pntd.0002544

**Published:** 2014-02-13

**Authors:** Xiang Y. Han, Francisco J. Silva

**Affiliations:** 1 Department of Laboratory Medicine, The University of Texas M. D. Anderson Cancer Center, Houston, Texas, United States America; 2 Institut Cavanilles de Biodiversitat i Biologia Evolutiva, Universitat de València, València, Spain; 3 CIBER en Epidemiología y Salud Pública, Madrid, Spain; Oxford University Clinical Research Unit, Viet Nam

## Abstract

Leprosy is a chronic infection of the skin and nerves caused by *Mycobacterium leprae* and the newly discovered *Mycobacterium lepromatosis*. Human leprosy has been documented for millennia in ancient cultures. Recent genomic studies of worldwide *M. leprae* strains have further traced it along global human dispersals during the past ∼100,000 years. Because leprosy bacilli are strictly intracellular, we wonder how long humans have been affected by this disease-causing parasite. Based on recently published data on *M. leprae* genomes, *M. lepromatosis* discovery, leprosy bacilli evolution, and human evolution, it is most likely that the leprosy bacilli started parasitic evolution in humans or early hominids millions of years ago. This makes leprosy the oldest human-specific infection. The unique adaptive evolution has likely molded the indolent growth and evasion from human immune defense that may explain leprosy pathogenesis. Accordingly, leprosy can be viewed as a natural consequence of a long parasitism. The burden of leprosy may have affected minor selection on human genetic polymorphisms.

## Leprosy As a Strictly Human Disease

Human beings have contracted leprosy for millennia, as documented in ancient cultures. A chronic infection of the skin and nerves, leprosy is caused by *Mycobacterium leprae* and the newly discovered *Mycobacterium lepromatosis* [1]

Rare infections in animals have been seen incidentally, such as in chimpanzees and monkeys [2], [3], and naturally, such as in armadillos in the southern United States [4], [5]. However, multilocus typing of the armadillo *M. leprae* strains suggests that they were of human origin for at most a few hundred years [6]. Thus, the animal likely first acquired the organism incidentally from early American explorers. Unfortunately, this incidental transmission was sustained in the armadillo population, and it is now transmitted back to humans, making leprosy a zoonotic disease [4]. In the Old World, where leprosy was first known, there are no armadillos.

Leprosy bacilli dwell strictly in the intracellular milieu of macrophages and nerve Schwann cells. Once out of the human body, they fail to grow on artificial media, unlike all other *Mycobacterium* species do (∼150 species). This cultivation difficulty has impeded research and care for the disease.

## Out of Africa with Leprosy

The age of human leprosy was further traced by analyzing the parasitic *M. leprae*. Genomes of four *M. leprae* strains from India, Thailand, Brazil, and the US have been sequenced in the past decade [7], [8]. The genome contains 3.3 megabase pairs (Mb), the smallest of the *Mycobacterium* species genomes, including *M. tuberculosis* (4.4 Mb) [9], *M. avium* (5.5 Mb) [10], *M. marinum* (6.6 Mb) [11], *M. ulcerans* (5.6 Mb) [12], and others. In addition, the *M. leprae* genome has undergone a reductive evolution, with ∼40% of the genes inactivated to become pseudogenes [7]. This is unique among pathogenic bacteria; among all other bacteria, only endosymbiont *Sodalis glossinidius* has ∼38% pseudogenes [13]. These genome features match this organism's strict intracellular nature.

Comparative analyses of the four *M. leprae* genomes have revealed only clonal differences of ∼200 bp or 0.005% of the 3.3 Mb [8]. Multilocus typing further showed clonality in 400 worldwide *M. leprae* strains. A few clonal patterns matched the global human migration routes during the past 100,000 years, suggesting that leprosy originated in Africa [6], [8]. These findings also demonstrate extraordinary stability of *M. leprae* in modern humans. All conclusions hold well in a new study of genome sequences of 12 additional *M. leprae* strains, including five medieval European strains (600–1,000 years old) and seven present time strains [14].

## *M. leprae* Evolution

The lean genome and the clonal stability suggest that leprosy's parasitism in humans dates back further than 100,000 years. Central to this date is when, where, and how the massive reductive evolution occurred.

Pseudogenes are considered to be molecular fossils. Gomez and colleagues [15] analyzed the ages of 611 *M. leprae* pseudogenes by comparing them with the corresponding orthologous genes from *M. tuberculosis*. They found a normal distribution of the ages, indicating that the massive gene inactivation occurred as a single continuous event, likely in one type of host or one environment. The estimated mean age of the pseudogenes, based on the mutation rates of *Escherichia coli*, was ∼9 million years (Myr); hence, the authors concluded that the reductive evolution took place in the past ∼20 Myr. These insightful findings thus trace the one-mode evolution from current human host to possibly early hominids.

What drove this reductive evolution? We reasoned earlier [15], [16] that the driving force was the adaptation from a free-living lifestyle, as all mycobacteria have except leprosy and tuberculosis bacilli, to an increasingly parasitic lifestyle in the host. During the past decade, comparative genomics have demonstrated that these changes in lifestyle lead to relaxation of natural selection and make many genes non-essential to initiate reductive evolution. In the early stages, many genes, usually those that are less selectively constrained, are inactivated [7], [13], [17], [18]. Such inactivation occurs through missense or nonsense nucleotide substitutions, insertions, or deletions or mobilization of insertion elements [19]. A tendency to lose nonfunctional DNA follows [20]–[22]. Eventually, the genome of a parasitic or symbiotic bacterium becomes smaller.

Table 1 compares genomes of parasitic *M. leprae* and *M. tuberculosis* with those of free-living *M. marinum*, *M. ulcerans*, and *M. avium*. The findings fit well the living style hypothesis. In addition, as part of the early-stage niche adaptation, the *M. ulcerans* genome has evolved from its ancestral genome of 6.6 Mb of *M. marinum* that shares 99% sequence identity to 5.6 Mb with 16% pseudogenes [12]. All free-living mycobacteria are less pathogenic.

**Table 1:** Genomes, living styles, and pathogenicity of five *Mycobacterium* species.

Feature	*M. leprae*	*M. tuberculosis*	*M. ulcerans*	*M. marinum*	*M. avium* 104
Genome					
References; year	[7], [8]; 2001, 2009	[9]; 1998	[12]; 2007	[11]; 2008	[10]; 2013
Mega-base pairs	3.27	4.41	5.63	6.64	5.48
G+C content (%)	57.8	65.6	65.5	65.7	69.0
No. genes	1,604	3,974	4,160	5,424	5,120
No. pseudogenes	1,116	17	771	65	Rare
Living style	Human parasite	Human parasite	Free-living	Free-living	Free-living
Cultivation in media	No	Yes	Yes	Yes	Yes
Pathogenicity	Moderate	High	Moderate	Low	Low
Main infection sites	Skin, nerves	Lungs, bone, etc	Skin (legs)	Skin	Opportunistic
Route of infection	Likely nasal mucosa	Airway transmission	Water insect bite	Wound exposure	Airway, GI tract

GI: gastrointestinal tract.

## New Leprosy Agent *M. lepromatosis*

A new leprosy agent, named *M. lepromatosis*, was discovered in 2008 in patients who died of diffuse lepromatous leprosy (DLL) [1]. DLL is a unique severe form of leprosy that has been endemic in Mexico and Costa Rica for more than a century [23]–[29]. The etiologic agent had been presumed to be *M. leprae*, but it was never studied beyond microscopy. We carried out analyses of six genes of this acid-fast bacillus [1]; the results revealed an overall 7.4% difference from *M. leprae*, suggesting a new species. Similar to *M. leprae*, *M. lepromatosis* did not grow in culture.

Two recent case studies independently corroborated this new cause of leprosy by gene analyses. Vera-Cabrera et al. [30] reported a case of DLL in a Mexican woman due to *M. lepromatosis*. These authors confirmed the new species and excluded the presence of *M. leprae* from biopsied skin tissue. Jessamine and colleagues [31] reported a case of lepromatous leprosy caused by *M. lepromatosis* in a native Canadian man. The patient had manifested polyneuropathy for two years before the onset of a skin rash that led to biopsies and the diagnosis.

In a systematic analysis of tissue specimens from 120 patients from Mexico with various clinical forms of leprosy, we confirmed and differentiated the etiologic mycobacteria in 87 cases [32]. Of these, *M. lepromatosis* caused 55 cases, *M. leprae* caused 18 cases, and both species caused 14 cases. *M. lepromatosis* caused not only all 13 DLL cases specifically but also caused more cases of lepromatous leprosy and other clinical forms of leprosy. The results of this study suggest that *M. lepromatosis* is likely the dominant cause of leprosy in Mexico and co-exists with *M. leprae* in endemic areas. Both organisms may also cause dual infections in patients.

In addition to the cases from Mexico and Canada, *M. lepromatosis* was identified in Singapore, across the Pacific Ocean [33]. In a recent study [34], we demonstrated that *M. lepromatosis* also caused severe leprosy reactions, another common clinical feature of the disease. Therefore, *M. lepromatosis* is the long-elusive second cause of leprosy.

## *M. lepromatosis* Phylogeny

A further analysis of 20 genes and pseudogenes from *M. lepromatosis* revealed an overall nucleotide difference of 9.1% from *M. leprae*, verifying a species-level divergence [16]. Remarkably, the pseudogenes differed by 20.9%, whereas the protein-coding genes differed overall by 6.9%; the 16S rRNA gene, most conserved among bacteria, differed by 2%. The functional categories of these genes and pseudogenes matched—i.e., genes to genes and pseudogenes to pseudogenes. This 9.1% sequence difference contrasts starkly with the clonal worldwide *M. leprae* strains.

**Figure 1:**
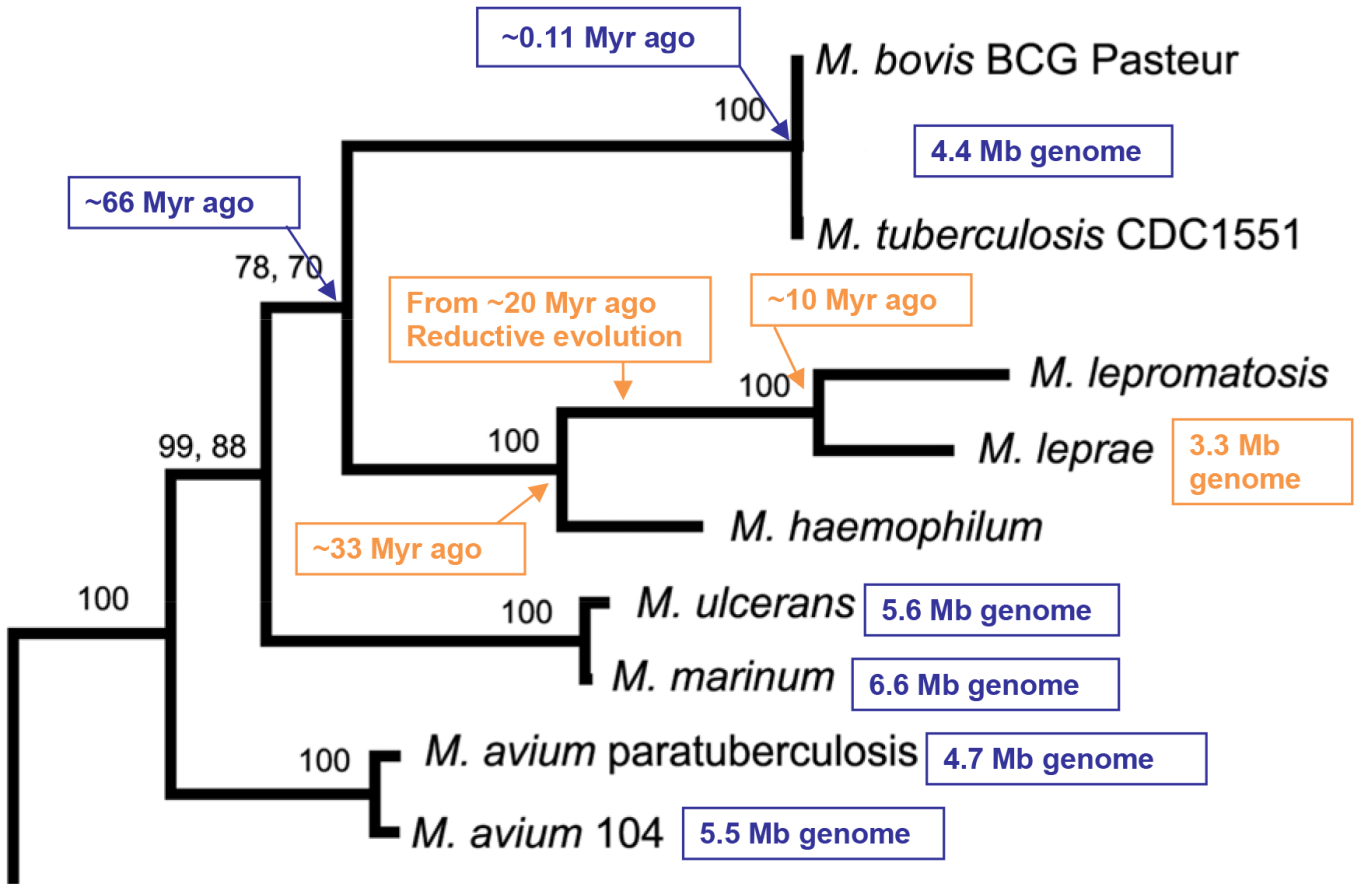
Phylogenetic tree of several *Mycobacterium* species based on the amino acid sequences of rpoB protein. Adapted from [15] **.**

In the study [16], we used conserved genes to construct a robust phylogenetic tree among several related *Mycobacterium* species (Figure 1); it revealed that *M. lepromatosis* and *M. leprae* diverged from the last common ancestor ∼10 Myr ago. This divergence occurred at or near the completion of the reductive evolution, as determined from the analysis of *M. leprae* pseudogenes mentioned above. Thus, *M. lepromatosis* is also an ancient organism despite its new recognition. The ages of the matched pseudogenes were also found to be similar, and common insertions and deletions were identified. These results indicate pseudogene status before divergence.

The newly published [14] *M. leprae* mutation rate of 6.13×10^−9^ substitutions per site per year allows us to estimate the divergence age, too. To do that, we compared the number of substitutions after the divergence of both species in all five pseudogenes and five conserved genes (total ∼12 kb) from the dataset [16]. We estimated the numbers of substitutions per site to be 0.00857, 0.15441, and 0.09245 for the nonsynonymous sites, synonymous sites, and in the pseudogenes, respectively. With the *M. leprae* coding density of 49.5% [7] and the fraction of nonsynonymous and synonymous sites derived previously [15], we came up the expected fraction of these sites to be 0.389, 0.106, and 0.505, respectively. Combining these fractions then produced an overall value of 0.06639 substitutions per site, which translates to 10.8 Myr given 6.13×10^−9^ substitutions per site per year. This divergence age is essentially identical to our previous estimate.

Together, the results of phylogenetic analyses suggest that the massive reductive evolution, ascribed to *M. leprae* previously, in fact occurred in the last common ancestor of both leprosy bacilli. Somehow, around the completion of this genome reduction, the ancestor, a specialized parasite, followed two separate evolutionary tracks, which led to the two species as we see at present. The Myr-long course resulted in much more variation in the pseudogenes than in the functional genes, suggesting again selection constraints for genes but not for pseudogenes.

## Leprosy versus Human Evolution

The evolution of leprosy bacilli occurred in two stages: the initial period up to 20 Myr ago, during which the last common ancestor underwent a massive reductive evolution to become a niche-adapted parasite with the smallest and least functional mycobacterial genome, and a second period ∼10 Myr to now, which began with the ancestor that followed two separate evolutionary paths; thus, two leprosy bacilli have maintained the leanest functional set of genes (genomes). During the past >100,000 years in the second stage, it is almost certain that they have been in humans, in view of the sole reservoir status, strict parasitism, and clonal stability during global human dispersals.

Human evolution also occurred during the past several Myr [35], [36]. In brief, starting from the superfamily *Hominoidae* (apes), the families *Hylobatidae* (gibbons) and *Hominidae* (great apes) diverged ∼18–20 Myr ago. *Hominidae* further evolved into the subfamilies *Homininae* (humans, gorillas, and chimpanzees) and *Ponginae* (orangutans) ∼15 Myr ago. Gorillas diverged from humans and chimpanzees ∼8–9 Myr ago, and humans and chimpanzees further separated ∼6–8 Myr ago. Finally, the human subtribe evolved through a few ancestral human genera, such as *Australopithecus anamensis*, *A. afarensis*, and *A. africanus* ∼2–5 Myr ago and *Homo habilis*, *H. erectus*, *H. rudolfensis*, *H. ergaster*, and other *Homo* species ∼2 Myr ago, to become the only extant *H. sapiens* today. Various *Homo* species have existed over the past ∼2 Myr [37].

As strict human parasites today, leprosy bacilli evolved over time and likely in space with humans. But at what point during human evolution did they jump to humans or our early ancestors? Here we propose the following hypotheses and examine them.

## Hypothesis 1: Single Mode Evolution in Hominid-Humans

The leprosy ancestor infected an ancestral ape species (up to ∼20 Myr ago) and began adapting from a free-living to a parasitic lifestyle. Over several Myr, a massive reductive evolution took place. At or near the completion of this evolution ∼10 Myr ago, the parasite, being carried in different host ape groups, evolved alongside the hosts, on separate evolutionary tracks. Over Myr, one host hominid lineage eventually evolved into modern humans, carrying with it one parasitic leprosy bacillus, either *M. leprae* or *M. lepromatosis*, and the other species was transmitted from another hominid species to pre-modern humans later (also Myr ago).

Alternatively, after reductive evolution in one ape species, the leprosy ancestor evolved into the lineages of *M. lepromatosis* and *M. leprae* (several Myr ago) and became two different species overtime. Most of the bacterial lineages derived from both species have been lost, along with many host ape species; we now have almost clonal *M. leprae* and *M. lepromatosis* in modern humans.

This hypothesis portrays a single mode continuum of the parasitic reductive evolution in the human lineage from hominids to *Homo*. The existence of many ancestral human genera and *Homo* species in the last several Myr does not contradict this possibility. Hence, even considering dating variations, at least one leprosy bacillus may have been with humans for millions of years.

The long time span raises another question: would the bacilli have been lethal enough to wipe out the host populations from hominids to modern humans? This is unlikely for the following reasons: (1) the last common ancestor of the leprosy bacilli likely had low pathogenicity to its hominid host before and during the reductive evolution, given that *Mycobacterium haemophilum*, an environmental organism that is closest to the leprosy ancestor in phylogeny (Figure 1) [16], [38], seldom causes disease in humans or animals; (2) the long incubation (up to decades) and chronicity of leprosy would be unlikely to halt host reproduction; and (3) most modern humans are not susceptible to the leprosy bacilli [29], probably because of innate immunity or genetic polymorphisms.

## Hypothesis 2: Incidental Transmission to Humans

Leprosy's ancestor and, later, the *M. leprae* and *M. lepromatosis* lineages evolved in diverse hosts (apes and otherwise) for Myr to become niche-adapted mature parasites. Then the two leprosy bacilli were transmitted to modern or near-modern humans incidentally and recently (a few hundred thousand years ago). This incidental nature is similar to the recent transmission of *M. leprae* between humans and armadillos. The leprosy bacilli thrived in modern humans as the population size and density expanded in favor of transmission.

The following facts, however, contradict this scenario. First, there are no other known reservoirs; nor are similar mycobacteria with lean genome and numerous pseudogenes found anywhere else. Second, *M. leprae* proliferates in immune-defective athymic mice, but barely in normal mice [39], suggesting vulnerability to host defense. This implies that the niche-adapted weakened bacillus, when first transmitted to humans, would unlikely escape human immunity to sustain transmission in the population. Third, if it survived human defense, the new adaptation would have led to, in a few hundred thousand years, substantial variations in the genome and a second mode in pseudogene age distribution; neither was found [8], [15]. Hence, an incidental nature of human leprosy is unlikely.

## Leprosy and Human Evolution

Many studies in the past decades have shown human genetic susceptibility to leprosy, and the clinical manifestations or severity have also been classified by immunity [40]. Table 2 summarizes the results of several large, recent studies that have demonstrated the effects of single nucleotide polymorphisms involving many genes, most notably HLA DR-DQ, NOD2, TNFSF15, TLR1, and IL23R [41]–[47]. In a genome-wide association study of Chinese populations, Zhang et al. [41], [42] found that 19 alleles were associated with risk for or protection against leprosy. Six of these alleles were also replicated, with similar effects, in a Vietnamese population [43]. In studies of Nepalese [44] and Indian populations [45], [47], seven alleles and one haplotype that links four nearby alleles were found to be associated. Of the Indian studies, one [45] also replicated several alleles that had been implicated in previous studies, and the other [47] found an association among the copy numbers of IL23R and analyzed haplotypic interactions of several new and previously identified alleles. A few of these studies also found associations between these alleles and leprosy reactions or severity.

**Table 2:** Recent studies of polymorphic single nucleotides in the human genome that are significantly associated with risk for or protection against leprosy.

Study population, year, reference	SNP, rs#	Chr	Gene	Allele and frequency		Major allele on leprosy		Note
				Major	Minor	Odds ratio	Effect	
Chinese, 2009 [41],	4574921	9	TNFSF15	A, 0.68	G, 0.32	0.76	Protective	
Same	6478108	9	TNFSF15	G, 0.54	A, 0.46	0.73	Protective	
Same	10114470	9	TNFSF15	A, 0.53	G, 0.47	0.78	Protective	
Same	10982385	9	TNFSF15	A, 0.56	C, 0.44	0.84	Protective	
Same	3088362	13	CCDC122	C, 0.74	A, 0.26	0.66	Protective	
Same	3764147	13	LACC1	A, 0.69	G, 0.31	0.60	Protective	
Same	3135499	16	NOD2	A, 0.79	G, 0.21	0.86	Protective	
Same	7194886	16	NOD2	G, 0.86	A, 0.14	0.61	Protective	
Same	8057341	16	NOD2	A, 0.78	G, 0.22	0.85	Protective	
Same	9302752	16	NOD2	A, 0.71	G, 0.29	0.63	Protective	
Same	602875	6	HLA-DR-DQ	A, 0.68	G, 0.32	1.49	Risk	
Same	40457	8	RIPK2	A, 0.72	G, 0.28	1.30	Risk	
Same	42490	8	RIPK2	G, 0.58	A, 0.42	1.32	Risk	
Same	1873613	12	LRRK2	A, 0.75	G, 0.25	1.16	Risk	
Same	9533634	13	CCDC122	A, 0.76	G, 0.24	1.32	Risk	
Same	10507522	13	LACC1	A, 0.69	G, 0.31	1.47	Risk	
Chinese, 2011 [42],	2275606	6	RAB32	A, 0.79	G, 0.21	0.77	Protective	
Same	16948876	16	CYLD	A, 0.97	G, 0.03	0.64	Protective	
Same	3762318	1	IL23R	G,0.90	A, 0.10	1.45	Risk	
Vietnamese, 2012 [43],	42490	8	RIPK2	A, 0.52	G, 0.48	0.83	Protective	Replicated
Same	3088362	13	CCDC122	C, 0.75	A, 0.25	0.74	Protective	Replicated
Same	3764147	13	LACC1	A, 0.62	G, 0.38	0.75	Protective	Replicated
Same	9302752	16	NOD2	A, 0.80	G, 0.20	0.79	Protective	Replicated
Same	602875	6	HLA-DR-DQ	A, 0.73	G, 0.27	1.61	Risk	Replicated
Same	10507522	13	LACC1	A, 0.74	G, 0.26	1.43	Risk	Replicated
Nepalese, 2010 [44],	12448797	16	NOD2	T, 0.96	C, 0.04	0.46	Protective	
Same	2287195	16	SLIC1	A, 0.73	G, 0.27	0.66	Protective	
Same	8044354	16	NOD2	A, 0.65	G, 0.35	0.65	Protective	
Same	1477176	16	CYLD	T, 0.85	C, 0.15	2.27	Risk	
Indian, 2010 [45],	1071630	6	HLA-DR-DQ	C, 0.50	T, 0.50	0.43	Protective	
Same	927650	6	HLA-DR-DQ	T, 0.73	C, 0.27	0.45	Protective	
Same	5743618	4	TLR1	A, 0.89	C, 0.11	3.23	Risk	Replicated
Turkish, 2007 [46],	5743618	4	TLR1	A, 0.57	C, 0.43	2.08	Risk	See text
Indian, 2013 [47],	4 rs haplo	5	IL12B	C, 0.34	A, 011	0.81	Protective	See text, below

Note: the alleles and frequencies were from uninfected controls. For consistency, the odds ratios for the infected and uninfected were aligned by the major allele. SNP: single nucleotide polymorphism; Chr: chromosome. Ref. [47] study: the haplotypes of four SNPs of rs2853694 (C), rs2853697 (A), rs3181216 (A), and rs3181225 (C) with a frequency of 0.34 were compared with their alternate three haplotypes, which had a combined frequency of 0.67.

Among all 34 significant associations listed in Table 2, dominant alleles or haplotype were observed more frequently with protection (22 of 34) than with risk (12 of 34), hinting at a possible selection or adaptation effect. For example, the risk allele G of rs40290 in gene RIPK2 carried a frequency of 0.58 in the Chinese but 0.48 in the Vietnamese [41], [43]; the protective allele C of rs5743618 in gene TLR1 was more common and highly differentiated outside of African populations, 0.11 in Indians, 0.43 in Turks, and even higher in Europeans [45], [46]. This allele caused an amino acid mutation, resulting in a functional change of the toll-like receptor that is likely involved in trafficking pathogenic microbes [46], [48]. Thus far, no alleles with contradictory findings have been noted, although studies have been unable to replicate the implicated alleles because of variations in disease prevalence and history, population allelic frequencies, and scales and method of studies.

Similar to leprosy, tuberculosis has been a major human disease for ∼100,000 years. Being more fatal than leprosy, tuberculosis has also been found to have some selective effects on human evolution (reviewed by Gagneux [49]). Some alleles, such as those in the genes HLA DR-DQ and NOD2, are implicated in both.

The results of these and many earlier genetic studies suggest that variations in humans' susceptibility to leprosy involve complex traits of numerous polymorphic alleles. Yet, the observed odds ratios in Table 2 are relatively small (i.e., mostly within 2-fold), suggesting minor selective effect. Hence, considering leprosy a genetic disease [50] is an exaggeration, as another author has noted [51]. The disease has one cause (two species of leprosy bacilli); without the cause, there is no disease.

## Insights into Leprosy Pathogenesis

Knowing the bacillary evolution may lend insights into leprosy pathogenesis. Clinically, leprosy begins insidiously and manifests a spectrum of disease from severe lepromatous leprosy to mild tuberculoid form. In lepromatous leprosy, particularly late stage, and in DLL caused by *M. lepromatosis* [1], [24], [28], the bacilli disseminate to organs and tissue in a massive load, known as globi [29]. Bacillemia also occurs, but more than half of the patients have little constitutional response, such as fever. In tuberculoid leprosy, the host immunity contains the bacilli [40]. These characteristics accord with low acute virulence of the lean indolent bacilli.

The heavy bacillary burden in lepromatous leprosy (and DLL) has been puzzling and interpreted since the 1960s, with revisions, as an antigen-specific weakness of the host immune system in tackling *M. leprae* [29], [40], [52]. The human immunodeficiency virus (HIV) pandemic since the 1980s has witnessed, in patients with late-stage HIV infections, similar disseminated *M. avium* infection, along with other opportunistic infections [53], [54]. In this setting, the weak pathogen *M. avium* proliferates uncontrollably because of the viral destruction of the host's immune cells. In leprosy, however, the host has almost intact immune function [29], [55], so how do the bacilli amass under immune radar? This paradox points to a bacillary immune evasion [55], [56], a notion cherished by immunologists and further supported by recent human genome studies of all polymorphic alleles (over a million) that showed no severe human defects, as discussed above.

In experimental animal models, *M. leprae* falls to mouse immunity [39], [52] and also mostly to cynomolgus monkey's [57]; how could it thwart human immunity instead? Here we ascribe origination of this immune tolerance or evasion to the multi-Myr parasitic evolution of the leprosy bacilli in the human lineage. Early in the course, the parasitic lifestyle was harsh because the ancestor of leprosy bacilli had to escape host immune attack. This drove adaptation of these hardy resilient bacilli, as all mycobacteria are well known for (such as tolerating 2% NaOH used to kill other bacteria in clinical specimens), to mutate or rid those immune-alarming proteins or molecules while retaining the protective ones. This adaptation, intracellular dwelling, and relaxation initiated the Myr reductive evolution to inactivate genes that were no longer needed. Eventually, a lean genome with least number of antigens and immunogenicity was molded specifically from this long chase–hide game. The organism also became an obligate human parasite. Thus, the host immunity drives a parasitic bacterial evolution; this differs from a symbiotic evolution in which the host tames and eventually assimilates the bacterium for mutual benefits.

In fact, the *M. leprae* genome predicts 1,604 proteins or antigens [7], which is only 40% of those predicted from the *M. tuberculosis* genome [9] and 31% of those from *M. avium* (Table 1) [10]. One class of antigens, the PE/PPE family proteins, that is abundant in *M. tuberculosis* with 167 genes [9], is rare in the *M. leprae* genome with only nine intact genes and 30 pseudogenes [7]. These PE/PPE proteins are possible sources of genetic and antigenic variations [9], [58]; many recent studies have further suggested that these proteins are at the host–pathogen interface [59]. A study of the PPE38 gene region of clinical *M. tuberculosis* strains has indicated a rapid evolution rate of these genes and predicted that their functional loss could aid immune evasion [60]. Therefore, as shown in Table 3, the parasitic evolution of *M. leprae* has likely eliminated most PE/PPE proteins preferentially to evade host immune attack.

**Table 3:** Preferential inactivation of the PE/PPE family genes during the reductive evolution of *M. leprae*.

Category	PE/PPE family	All others	Total
No. pseudogenes (%)	30 (1.10)	1,086 (39.9)	1,116 (41.0)
No. genes (%)	9 (0.33)	1,595 (58.6)	1,604 (59.0)
Total (%)	39 (1.43)	2,681 (98.6)	2,720 (100)
Odds ratio of PE/PPE gene inactivation = 4.9, χ^2^ = 21.1, df = 1, p<0.0001			

*M. leprae* vs *M. tuberculosis*: % PE/PPE genes, 9 of 1,604 versus 167 of 3,974, odds ratio = 0.13, χ^2^ = 49.6, p<0.0001; % all PE/PPE genes and pseudogenes, 39 of 2,720 versus 167 of 3,974, odds ratio = 0.33, χ^2^ = 41.5, p<0.0001.

Data from [7] and [9].

*M. leprae* also amply produces phenol glycolipid-1 (PGL-1), a cell wall component [61]. PGL-1 elicits humoral response in patients, but hardly cellular immunity that actually controls leprosy [55], [62]. The antibody level to PGL-1 parallels bacillary burden as a diagnostic marker of lepromatous leprosy [62]. Functionally, PGL-1 aids bacillary invasion into Schwann cells [55], which in turn activates the cells to further spread the bacilli [63]. Studies have also shown convincingly that PGL-1 suppresses or subverts immune defense [64]–[68]. Thus, PGL-1 shields and perpetuates the parasitism. Six genes, likely involved in PGL-1 synthesis, have been identified, without finding of related pseudogenes [65], suggesting conservation despite genome reduction. In a fatal DLL case caused by *M. lepromatosis*, the PGL-1 antibody titer was also found to be strong [1], hinting abundant PGL-1 production by this species, too. Finally and above all, the lipid-rich cell wall of the leprosy bacilli, apparent from acid-fast stains and microscopy, the defining feature of mycobacteria, and the strongest defense wall among all bacteria (nearly 10,000 species), remains intact after reductive evolution. Therefore, it is likely that the lipid-rich cell wall has protected the leprosy bacilli from host clearance and enabled the unique Myr-long parasitic evolution.

The immune evasion is likely a gradual and dynamic process with a delicate balance between the bacilli and immunity. If the evasion gains, the disease worsens. If the evasion falls, the immunity prevails. For instance, in patients with borderline lepromatous leprosy, the disease is unstable and may progress towards the severe lepromatous form with increasing bacillary load. When antimicrobial therapy kills the bacilli, the immunity recovers, leading to inflammatory response known as leprosy reactions, which usually occur after months of treatment [29], [55]. Notably, the complexity of immune evasion in leprosy requires more studies to refine the details. Linking it with the parasitic adaptive evolution of the bacilli in this proposal unifies its role in pathogenesis, its origin, and its specificity to the human immune system.

## Conclusion

In summary, the leprosy bacilli *M. leprae*, *M. lepromatosis*, and their last common ancestor most likely evolved, both in time and space, with humans. They gradually settled in humans or early hominids millions of years ago as obligate intracellular parasites. This makes leprosy the oldest human-specific infection. The unique parasitic evolution may be a key piece in solving the puzzle of leprosy pathogenesis. Accordingly, leprosy can be viewed as a natural consequence of a long parasitism. The long burden of leprosy may have exerted minor selection on human genetic polymorphisms.

**Key Learning Points**

- Evolutionary origin and approximate timing of the leprosy bacilli *M. leprae*, *M. lepromatosis*, and their most recent common ancestor
- How to reconcile the parasitic evolution of the leprosy bacilli with their current human host and the most likely, remote past human-hominid host(s)
- Human genetic polymorphisms and minor susceptibility to leprosy infection.
- Evidence of the parasitic evolution of the bacilli and adaptation to host milieu in explanation of leprosy pathogenesis

**Five Key Papers in the Field**

1. Cole ST, Eiglmeier K, Parkhill J, James KD, Thomson NR, et al. (2001) Massive gene decay in the leprosy bacillus. Nature 409:1007–1011.
2. Monot M, Honoré N, Garnier T, Zidane N, Sherafi D, et al. (2009) Comparative genomic and phylogeographic analysis of *Mycobacterium leprae*. Nat Genet 41: 1282–1289.
3. Han XY, Seo Y-H, Sizer KC, Schoberle T, May GS, et al. (2008) A new *Mycobacterium* species causing diffuse lepromatous leprosy. Am J Clin Pathol130: 856–864.
4. Gomez-Valero L, Rocha EPC, Latorre A, Silva FJ (2007) Reconstructing the ancestor of *Mycobacterium leprae*: The dynamics of gene loss and genome reduction. Genome Res 17: 1178–1185.
5. Han XY, Sizer KC, Thompson EJ, Kabanja J, Li J, et al. (2009) Comparative sequence analysis of *Mycobacterium leprae* and the new leprosy-causing *Mycobacterium lepromatosis*. J Bacteriol 191: 6067–6074.
